# Antibiotic resistance spectrums of *Escherichia coli* and *Enterococcus* spp. strains against commonly used antimicrobials from commercial meat-rabbit farms in Chengdu City, Southwest China

**DOI:** 10.3389/fvets.2024.1369655

**Published:** 2024-05-02

**Authors:** Chen Sun, Ziye Wang, Yan Li, Jian Huang

**Affiliations:** ^1^College of Animal Husbandary and Veterinary Medicine, Southwest Minzu University, Chengdu, China; ^2^Institute of Qinhai-Tibetan Plateau, Southwest Minzu University, Chengdu, China

**Keywords:** antimicrobial resistance, antibiotics use, healthy meat-rabbits, epidemiological surveillance, ARG distribution

## Abstract

Antimicrobial resistance (AMR) is commonly associated with the inappropriate use of antibiotics during meat-rabbit production, posing unpredictable risks to rabbit welfare and public health. However, there is limited research on the epidemiological dynamics of antibiotic resistance among bacteria indicators derived from local healthy meat-rabbits. To bridge the knowledge gap between antibiotic use and AMR distribution, a total of 75 *Escherichia coli* (*E. coli*) and 210 *Enterococcus* spp. strains were successfully recovered from fecal samples of healthy meat-rabbits. The results revealed that diverse AMR phenotypes against seven commonly used antimicrobials, including ampicillin (AMP), amoxicillin-clavulanic acid (A/C), doxycycline (DOX), enrofloxacin (ENR), florfenicol (FFC), gentamicin (GEN), and polymycin B (PMB), were observed among most strains of *E. coli* and *Enterococcus* spp. in two rabbit farms, although the distribution pattern of antibiotic resistance between young and adult rabbits was similar. Among them, 66 *E. coli* strains showed resistance against 6 antimicrobials except for PMB. However, 164 *Enterococcus* spp. strains only exhibited acquired resistance against DOX and GEN. Notably, the DOX-based AMR phenotypes for *E. coli* and *Enterococcus* spp. strains were predominant, indicating the existing environmental stress conferred by DOX exposure. The MICs tests suggested elevated level of antibiotic resistance for resistant bacteria. Unexpectedly, all GEN-resistant *Enterococcus* spp. strains resistant high-level gentamicin (HLGR). By comparison, the *blaTEM, tetA, qnrS* and *floR* were highly detected among 35 multi-resistant *E. coli* strains, and *aac[6']-Ie-aph[2']-Ia* genes widely spread among the 40 double-resistant *Enterococcus* spp. strains. Nevertheless, the presence of ARGs were not concordant with the resistant phenotypes for a portion of resistant bacteria. In conclusion, the distribution of AMR and ARGs are prevalent in healthy meat-rabbits, and the therapeutic antimicrobials use in farming practice may promote the antibiotic resistance transmission among indicator bacteria. Therefore, periodic surveillance of antibiotic resistance in geographic locations and supervisory measures for rational antibiotic use are imperative strategies for combating the rising threats posed by antibiotic resistance, as well as maintaining rabbit welfare and public health.

## 1 Introduction

In recent years, the escalating public concerns over the evolution and spread of bacteria resistance between livestock and humans have underscored the need for surveillance on the judicious use of antibiotics in food-producing animals (1–3). However, the aggressive use of prophylactic and therapeutic antimicrobials during intensive rabbit production may facilitate population-level transfer of antibiotic resistance in farming environments (4, 5). These inadvertent consequences could potentially expedite the acquisition of antibiotic resistance and virulence among commensal bacteria in gastrointestinal tract (6), such as *Enterobacter* spp. and *Enterococcus* spp. (7), thereby contributing to gut microbiota dysbiosis and intestinal barrier impairments (8). Several genotypes of opportunistic *E. coli* or *Enterococci* strains have been confirmed as causative agents in newborn or weaning rabbits with diarrhea (9, 10), which urge us to explore the emergence and distribution of antibiotic resistance among the bioindicators derived from healthy rabbit population.

Although the restriction on the use of antibiotic feed additives as growth promoter in food-producing animals has significantly reduced the burden of bacterial antimicrobial resistance (AMR) on food-chains and ecosystems (11, 12), the inappropriate use of therapeutic antibiotics, such as tetracyclines, chloramphenicols and β-lactams (4, 5), remains an overlooked attention to the transferable antibiotic resistance resulting from antibiotics stress or bacteria contamination in food animal production systems. Since the emerging “superbugs” that exhibit resistance to critically important antimicrobials (CIAs), such as extended spectrum beta-lactamases (ESBL) (like *blaTEM, blaCTX-M, blaSHV)*, as well as phosphoethanolamine (pEtN) transferase (like *mcr-1*and *mcr-2)* producing *E. coli* strains, high-level gentamicin-resistant (HLGR) and vancomycin-resistant *Enterococcus* spp. strains, have been frequently reported in domestic animals (13–17), the relationship between antibiotic use and antibiotic resistance distribution should be continously monitored, particularly in the case of neglected healthy farmed rabbits.

Limited epidemiological studies have reported the presence of antibiotic-resistant *E. coli* and *Enterococcus* spp. isolates in domestic or wild rabbit populations (18–21), exhibiting diverse AMR spectrums and distinctive ARG distribution across different geographical areas. However, there is a lack of knowledge regarding updated epidemiological data in Chengdu City, which is one of largest markets for meat-rabbit production and consumption in Southwest China. Therefore, this investigation aims to characterize the antibiotic resistance in indicator *E. coli* and *Enterococcus* spp. strains derived from healthy meat-rabbits. The findings will enhance our understanding of the emergence of antibiotic resistance conferred by routinely used therapeutic antibiotics in a context of reduced antibiotic use and residues in food animals, thereby raising awareness about rational antibiotic use to combat the rising threats posed by antibiotic resistance in rabbit farming practices.

## 2 Materials and methods

### 2.1 Ethics approval and consent to participate

All methods involving the animal sampling and handling followed the criteria of animal welfare regulation. Informed consent was obtained from commercial rabbit farm owners, and the research protocol was approved by the Research Ethics Committee of Southwest Minzu University (approval number no. 2021-MDLS-037).

### 2.2 Sampling and bacteria isolation

Two hundred and forty-seven freshly voided fecal samples were randomly collected from 118 young (< 8 weeks) and 129 adult healthy rabbits (> 24 weeks) raised in two large-scale rabbit farms situated in suburbs of Chengdu City, Southwest China, from April to June, 2021. The samples were sterilely pooled in the plastic bags and stored at −80°C pending processing. Fecal samples were homogenized in 0.9% saline with a 1:2 ratio, then the diluted samples were inoculated into Luria-Bertani (LB) and Tryptic soy agar (TSA) (Hopebiol, Qingdao, China), respectively, and incubated in thermostatic incubator at 37°C for 24 h in aerobic conditions. The non-repetitive single colony per sample was cultured on Eosin methylene blue (EMB) agar and *Enterococcus* agar (Hopebiol, Qingdao, China) to preliminarily differentiate the *E. coli* or *Enterococcus* spp. isolates, respectively. After that, the presumptive bacteria isolates were further purified for later molecular identification.

### 2.3 DNA extraction and 16S rRNA gene analysis

The bacterial DNA was extracted using phenol-chloroform protocol (Tiangen, Beijing, China) as instruction described. The PCR assay targeting highly conserved 16S rRNA gene fragment was applied to test all suspected isolates. The primers 27F-ATGGCTCAGATTGAACG and 1492R-CAGGTTCCCCTACGGTTA were used for batcerial identification as described in the literature (22). Reaction system: 2 × Taq PCR MasterMix II (10 μM) (Tiangen, Beijing, China), 12.5 μL, 27F and 1492R primers (10 μM) 0.5 μL each, 1 μL of DNA template, and ddH_2_O replenished the system to 25 μL. PCR reaction conditions: pre-denaturation temperature of 94 °C for 3 min, denaturation temperature of 94 °C for 30 s, annealing and restitution temperature of 55 °C, PCR reaction conditions: pre-denaturation at 94°C for 3 min, denaturation at 94°C for 30 s, annealing at 55°C for 30 s, extension at 72°C for 1 min and 30 s for 30 cycles, and final extension at 72°C for 5 min on veriti 96-well thermal cycler (Thermo Fisher Scientific, USA). The 5 μL of each reaction were analyzed on 1% (w/v) agarose gel and submitted for DNA sequencing (Sangon Biotech, Shanghai, China). The species identification of *E. coli* and *Enterococcus* spp. isolates was accomplished by nucleotide sequence alignments on BLAST (https://blast.ncbi.nlm.nih.gov/Blast.cgi).

### 2.4 Antibiotic susceptibility testing

The availablility of therapeutic antibiotics were confirmed by the farm owners, and 7 commonly used antimicrbiobials including ampicillin (AMP), amoxicillin-clavulanate (A/C), doxycycline (DOX), enrofloxacin (ENR), gentamicin (GEN), florfenicol (FFC) and polymyxin B (PMB), were prepared in injectable or oral formulations. These antimicrobials are routinely or sporadically used in the two rabbit farms for suspected bacterial infection. Herein, the antibiotic susceptibility of bacteria isolates were tested for AMP, A/C, DOX, ENR, GEN, FFC and PMB using Kirby-Bauer (K-B) agar disk diffusion method (Hangzhou microbial reagent CO., LTD, Hangzhou, China). Then, the minimal inhibitory concentrations (MICs) of these antimirobials (Meilunbio, Dalian, China) for antibiotic-resistant *E.coli* and *Enterococcus spp*. strains were further analyzed by microbroth dilution method according to the recommendation of Clinical and Laboratory Standards Institute (CLSI, 2018) (23). In addition, high-level GEN-resistant (HLGR) *Entercococcus* spp. strains (MIC ≥ 500 μg/mL) were determined according to the recommended screen test (CLSI, 2014) (24). *E. coli* ATCC 25922 and *Enterococcus faecalis* ATCC29212 were used as quality control strains.

### 2.5 Antibiotic resistance genes detection

Based on the AMR phenotypes of multi-resistant *E.coli* or double-resistant *Enterococcus* spp. strains, representative antibiotic resistance genes (ARGs) that belonged to β-lactams (*blaTEM*), tetracyclines (*tetA, tetB, tetM*), fluroquinoloncs (*qnrD, qnrS*), chloramphenicols (*fexA, floR*), and aminoglycosides (*aac[6']-Ib* for *E. coli* strains*, aac[6']-Ie-aph[2”]-Ia* for HLGR *Enterococcsu* spp. strains) were detected using established PCR methods for these target genes (9, 25–27) (Supplementary Table 1.).

### 2.6 Data processing and analysis

The frequencies and percentages of all categorical variables were tabulated. The statistical significance (*P* < 0.05) was determined using Fisher's exact test or chi-squared tests in SPSS 18.0 (IBM Armonk Corp., NY, United States). The descriptive histograms were achieved using Graphpad Prism 8.0 (Graphpad Software Inc, San Diego, United States).

## 3 Results

### 3.1 Bacterial differentiation and identification

Totally, 75 suspected isolates of *E. coli* and 210 *Enterococcus* spp. were preliminary identified from 247 fecal samples by differential culture. The Gram-negative *E. coli* strains appeared to be rod-like bacillus and formed deep purplish-black colonies on EMB agar (Supplementary Figures 1A, C). On the other hand, the Gram-positive *Enterococcus* spp. strains appeared to be a spherical morphology and formed black colonies on *Enterococci* differential medium (Supplementary Figures 1B, D). DNA sequencing and molecular alignment further confirmed all suspected *E. coli* or *Enterococcus spp*. strains. In addition, the *Enterococcus* spp. isolates consisted of 142 *Enterococcus faecalis* (*E. faecalis*), 47 *Enterococcus faecium* (*E. faecium*), 15 *Enterococcus gallinarum* (*E. gallinarum*), and 6 *Enterococcus hirae* (*E.hirae*) strains. Among them, 51 *E. coli* strains (27 from young rabbits and 24 from adult rabbits) and 82 *Enteroccocus* spp. strains (45 from young rabbits and 37 from adult rabbits) were recovered from farm A, while 24 *E. coli* strains (9 from young rabbits and 15 from adult rabbits) and 128 *Enteroccocus* spp. strains (54 from young rabbits and 74 from adult rabbits) were recovered from farm B (Table 1).

**Table 1:** AMR frequencies of *E. coli* and Enterococcus spp. isolates from farming rabbits against commonly used antibiotics using the K-B method.

**Bacteria**	**Age**	**Antimicrobial agents**	**Farm A n/n (%)**	**Farm B n/n (%)**	***P*-value**
		Ampicillin (AMP)	20/27 (74.1)	2/9 (22.2)	0.014
		Amoxicillin-clavulanic acid (A/C)	9/27 (33.3)	1/9 (11.1)	0.392
		Doxycline (DOX)	17/27 (70.0)	9/9 (100.0)	0.039
	Young rabbits (*n =* 36)	Enrofloxacin (ENR)	12/27 (44.4)	1/9 (11.1)	0.114
		Florfenicol (FFC)	2/27 (8.3)	8/9 (88.9)	0
		Gentamicin (GEN)	1/27 (3.7)	8/9 (88.9)	0
*E. coli* (*n =* 75)		Polymycin B (PMB)	0/27 (0)	0/9 (0)	NC
		Ampicillin (AMP)	14/24 (58.3)	4/15 (26.7)	0.098
		Amoxicillin-clavulanic acid (A/C)	9/24 (37.5)	1/15 (6.67)	0.057
		Doxycline (DOX)	16/24 (66.7)	12/15 (80.0)	0.477
		Enrofloxacin (ENR)	5/24 (20.8)	2/15 (13.3)	0.686
	Adult rabbits (*n =* 39)	Florfenicol (FFC)	5/24 (20.8)	7/15 (46.7)	0.153
		Gentamicin (GEN)	0/24 (0)	8/15 (53.3)	0
		Polymycin B (PMB)	0/24 (0)	0/15 (0)	NC
		Ampicillin (AMP)	0/45 (0)	0/54 (0)	NC
		Amoxicillin-clavulanic acid (A/C)	0/45 (0)	0/54 (0)	NC
		Doxycline (DOX)	26/45 (57.8)	22/54 (40.7)	0.091
	Young rabbits (*n =* 99)	Enrofloxacin (ENR)	0/45 (0)	0/54 (0)	NC
		Florfenicol (FFC)	0/45 (0)	0/54 (0)	NC
Enterococcus spp. (*n =* 210)		Gentamicin (GEN)	11/45 (24.4)	29/54 (53.7)	0.003
		Polymycin B (PMB)	IR	IR	NC
		Ampicillin (AMP)	0/37 (0)	0/74 (0)	NC
		Amoxicillin-clavulanic acid (A/C)	0/37 (0)	0/74 (0)	NC
		Doxycline (DOX)	36/37 (97.3)	56/74 (75.68)	0.004
	Adult rabbits (*n =* 111)	Enrofloxacin (ENR)	0/37 (0)	0/74 (0)	NC
		Florfenicol (FFC)	0/37 (0)	0/74 (0)	NC
		Gentamicin (GEN)	7/37 (18.9)	17/74 (23.0)	0.625
		Polymycin B (PMB)	IR	IR	NC

n/n, number of resistant strains/total number of bacteria; NC, not computed; IR, intrinsic resistance.

### 3.2 Antibiotic resistance distribution and phenotypes

All bacteria strains were tested for their antibiotic resistance phenotype against seven commonly used antimicrobials using the K-B method. Among them, majority of *E. coli* strains exhibited resistance to DOX (54/75, 72.0%), AMP (40/75, 53.3%), FFC (22/75, 29.3%), A/C (20/75, 26.7%), ENR (20/75, 26.7%), and GEN (17/75, 22.7%), respectively. However, all *E. coli* strains were susceptible to PMB. Furthermore, all *Enterococcus* spp. strains showed sensitivity to A/C, AMP, FFC, and ENR while possessing intrinsic resistance (IR) to PMB. Notably, a significant number of resistant *Enterococcus* spp. strains against DOX (140/210, 66.7%) and GEN (64/210, 30.5%) were detected (Table 1).

**Figure 1:**
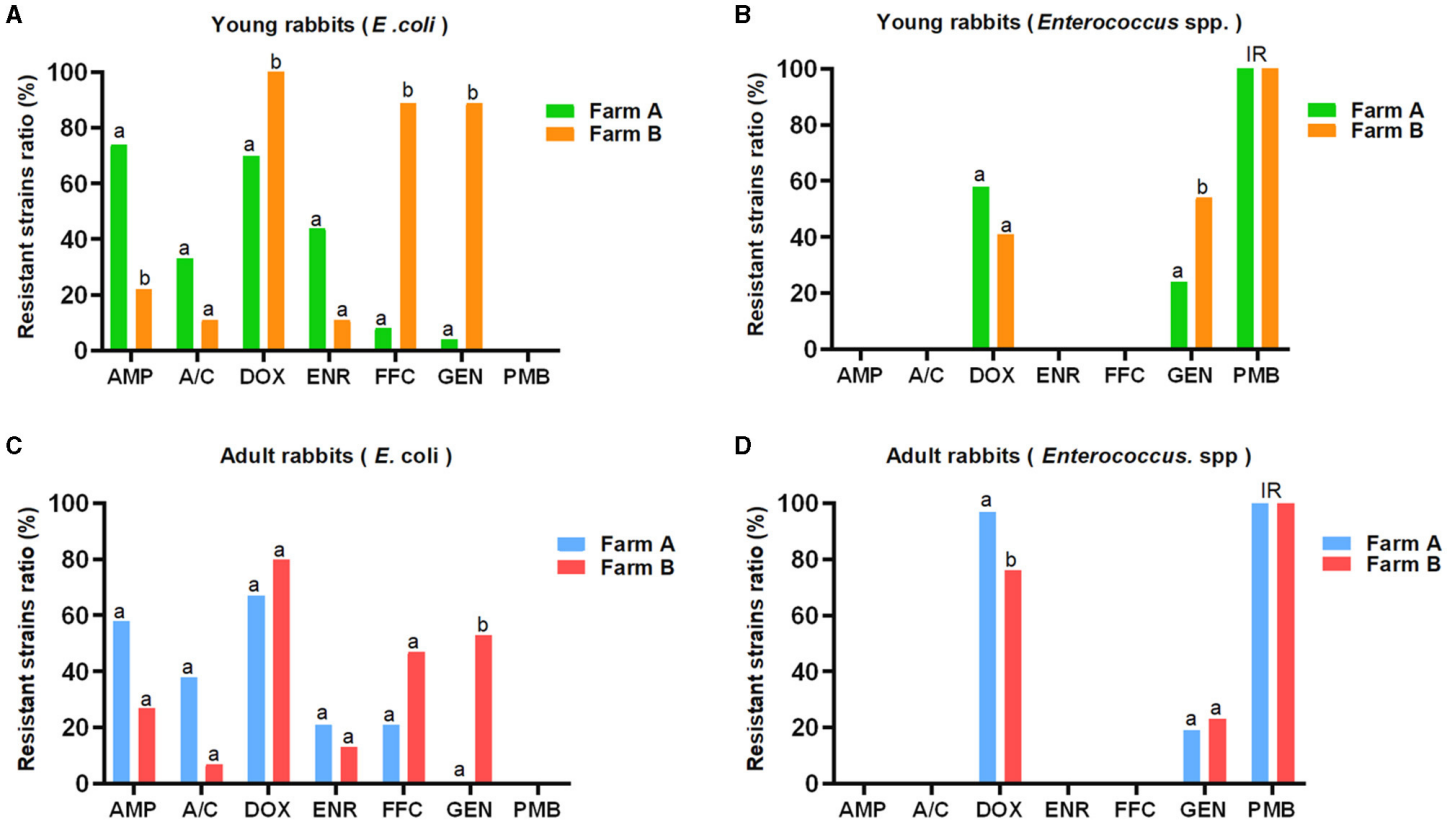
AMR distribution among *E. coli* and *Enterococcus* spp. strains derived from young and adult rabbits against seven commonly used antibiotics (K-B method). The *E. coli* **(A, C)** and *Enterococcus* spp. **(B, D)** strains showed distinct resistant rates against seven commonly used antibiotics. The prevalence of resistant *E. coli* strains from farm A was higher than that from farm B with respect to AMP, A/C, and ENR. However, it was lower for DOX, FFC, and GEN, respectively **(A, C)**. Similarly, the rate of DOX-resistant *Enterococcus* spp. strains from farm A exceeded that from farm B, but was lower for GEN **(B, D)**. Although the distribution patterns of antimicrobial resistance between young and adult rabbits were similar, there were no consistent statistical differences observed in terms of resistant *E. coli* or *Enterococcus* spp. strains for most antimicrobials. IR: intrinsic resistance. a, b: different letters mark statistical significance between variables.

In contrast, the prevalence of resistant *E. coli* strains against AMP (34/51, 66.7%), A/C (18/51, 35.3%), and ENR (17/51, 33.3%) from farm A were higher compared to farm B (AMP, 6/24, 25.0%; A/C, 2/24, 8.3%; ENR, 3/25, 12.0%), respectively. Conversely, the DOX-, FFC-, and GEN-resistant *E. coli* strains were more frequently observed in farm B (DOX, 21/24, 87.5%; FFC, 15/24, 62.5%; GEN, 16/24, 66.7 %) than farm A (DOX, 33/51, 64.7%; FFC, 7/51, 13.7%; GEN, 1/51, 2.0 %). Moreover, the proportion of DOX-resistant *Enterococcus* spp. strains from farm A (62/82, 75.6%) was higher than that from farm B (78/128, 60.9%), while the occurrence of GEN-resistant strains was lower in farm A (18/82, 22.0%) compared to farm B (46/128, 35.9%). The distribution pattern of antibiotic resistance among *E. coli* or *Enterococcus* spp. strains between young and adult rabbits was similar in two farms, but the statistical differences for the ratios of resistant bacteria against certain antimicrobials were not always concordant (Figure 1 and Table 1).

To investigate the AMR profiles of *E. coli* and *Enterococcus* spp. strains we further characterized the antibiotic resistance phenotypes of each bacteria (Table 2). Diverse AMR profiles were shown among resistant *E. coli* strains with single, double, and multiple resistance phenotypes against six commonly used antimicrobials, except for PMB. However, only single and double resistance phenotypes against DOX and GEN were found among *Enterococcus* spp. strains. Notably, the DOX-based AMR phenotypes in *E. coli* and *Enterococcus* spp. strains were most predominant. Additionally, a subset of multi-resistant *E. coli* (35/66, 53.0%) and double-resistant *Enterococcus* spp. strains (40/164, 24.4%) contributed to the diversity of AMR phenotypes.

**Table 2:** AMR profile of *E. coli* and *Enterococcus* spp. sfrains from farmed rabbits by K-B method.

**Bacteria**	**AMR phenotype**	**Number of isolates (%)**
	AMP	12 (16.0)
	DOX	9 (12.0)
	AMP-DOX	2 (2.7)
	DOX-ENR	3 (4.0)
	DOX-FFC	1 (1.3)
	A/C-AMP-DOX	4 (5.3)
	AMP-DOX-ENR	2 (2.7)
*E. coli* (*n =* 75)	AMP-DOX-FFC	1 (1.3)
	AMP-DOX-GEN	1 (1.3)
	DOX-FFC-GEN	12 (16.0)
	A/C-AMP-DOX-ENR	8 (10.7)
	A/C-AMP-DOX-FFC	3 (4.0)
	A/C-AMP-DOX-GEN	1 (1.3)
	AMP-DOX-ENR-GEN	2 (2.7)
	DOX-ENR-FFC-GEN	1 (1.3)
	A/C-AMP-DOX-ENR-FFC	4 (5.3)
	Susceptible to all tested antimicrobials	9 (12.0)
	DOX	100 (47.6)
*Enterococcus* spp. (*n =* 210)	GEN	24 (11.4)
	DOX-GEN	40 (19.1)
	Susceptible to all tested antimicrobials	46 (21.9)

### 3.3 The MICs of antibiotic-resistant *E. coli* strains and *Enterococcus* spp. strains

The MICs of resistant bacteria were determined using the microbroth dilution method, which yielded consistent results with the K-B method. Resistant strains of *E. coli* and *Enterococcus* spp. exhibited elevated levels of antibiotic resistance, as indicated by increased MIC values. Furthermore, all GEN-resistant *Enterococcus* spp. strains (64/64, 100%) demonstrated a high-level gentamicin resistance, whereas this phenomenon was not observed among GEN-resistant *E. coli* strains (0/17, 0%) (Table 3).

**Table 3:** Minimal inhibitory concentrations (MICs) of antibiotic-resistant *E. coli* and *Enterococcus* spp. strains.

**Bacteria**	**Antimicrobial agents (n)**	**MICs (**μ**g/mL)**							
		**8**	**16**	**32**	**64**	**128**	**256**	**>256**	**R Break point**
	Ampicillin (AMP) (*n =* 40)	0	0	2	9	4	0	25	≥32
	Amoxicillin-clavulanic acid (A/C) (*n =* 20)	1	13	6	0	0	0	0	≥8
*E. coli*	Doxycline (DOX) (*n =* 54)	0	6	44	4	0	0	0	≥16
	Enrofloxacin (ENR) (*n =* 20)	8	0	1	1	9	0	1	≥2
	Florfenicol (FFC) (*n =* 22)	0	0	0	1	1	1	19	≥32
	Gentamicin (GEN) (*n =* 17)	0	0	2	8	7	0	0	≥16
		**16**	**32**	**64**	**—**	**512**	**1024**	**2048**	**R Break point**
*Enterococcus* spp.	Doxycline (DOX) (*n =* 140)	49	89	2	—	NT	NT	NT	≥16
	Gentamicin (GEN) (*n =* 64)	0	0	0	—	35	20	9	HLGR≥500

n, number of resistant strains against specific antimicrobial; R, resistant; NT, not tested; HLGR, high-level gentamicin resistant.

### 3.4 Antibiotic resistance gene distribution among resistant bacteria

Based on the AMR phenotypes, a total of 35 multi-resistant *E. coli* strains and 40 double-resistant *Enterococcus* spp. strains (*n* = 40) were selected for further screening of relevant ARGs. Among the multi-resistant *E.coli* strains, *blaTEM* (97.1%, 34/35), *tetA* (94.3%, 33/35), *tetB* (25.7%, 9/35), *tetM* (2.9%, 1/35), *qnrS* (71.43%, 25/35), *floR* (57.14%, 20/35), and *aac(6')-lb* (11.4%, 4/35) genes were widely detected, respectively. Accordingly, infrequent distributions of *tetM* (25.0%, 10/40), *fexA* (10.0%, 4/40), and *floR* (7.5%, 3/40) were also observed among double-resistant *Enterococcus* spp. strains, while the *aac[6']-Ie-aph[2']-Ia* (77.5%, 31/40) gene was highly prevalent. Comparatively, *blaTEM, tetA, qnrS*, and *floR* genes for multi-resistant *E. coli* strains and the *aac[6']-Ie-aph[2”]-Ia* gene for double-resistant *Enterococcus* spp. strains were overrepresented (over 50%). However, the presence of ARGs were not consistent with the AMR phenotypes for a portion of resistant strains (e.g., *fexA* and *floR* genes were detected in DOX/GEN-resistant *Enterococcus* spp. strains) (Table 4).

**Table 4:** Antibiotic resistance genes (ARGs) of multi-resistance *E. coli* and double-resistant *Enterococcus* spp. isolates from healthy rabbits.

**Bacteria**	**Group of antibiotics**	**ARGs**	**Numbers of isolates (%)**
	β-lactams	bla_TEM_	34 (97.1)
		tetA	33 (94.3)
	Tetracyclines	tetB	9 (25.7)
		tetM	3 (25.7)
*E. Coli* (*n =* 35)	Fluroquinoloncs	qnrD	0 (0)
		qnrS	25 (71.4)
	Chloramphenicols	fexA	0 (0)
		floR	20 (57.4)
	Aminoglycosides	aac[6′]-Ib	4 (11.4)
		tetA	0 (0)
	Tetracyclines	tetB	0 (0)
		tetM	10 (25)
Enterococcus spp. (*n =* 40)	Chloramphenicols	fexA	4 (10)
		floR	3 (7.5)
	Aminoglycosides	aac[6']-Ie-aph[2”]-Ia	31 (77.5)

## 4 Discussion

It is widely recognized that symbiotic *E. coli* and *Enterococcus* spp. play a crucial role in maintaining intestinal homeostasis and integrity (28). However, they are also prone to acquiring antibiotic resistance and harboring antimicrobial resistance genes (ARGs) when repeatedly exposed to antibiotics, particularly in intensive food-animal husbandry (6, 7). Moreover, these antibiotic-resistant commensal bacteria have been reported as opportunistic pathogens causing diarrhea among rabbits (9, 10), and they even act as negative mediators to shield the antibiotic-sensitive pathogens during antibiotic treatment (29). Therefore, it is imperative to conduct surveillance on these bioindicators to assess the potential challenge posed by transferable antibiotic resistance for animal health.

Considering that commercial meat-rabbits may receive prophylactic or therapeutic antimicrobials to address suspected bacterial infection, such as respiratory or gastrointestinal infectious diseases, either through direct contact or environmental contamination (5, 30), which may significantly changes the composition of gastrointestinal microbiota (31) and antimicrobial resistance (AMR) phenotypes of commensal *E. coli* and *Enterocuccus* spp. strains (32). In this study, we successfully isolated two bacteria species from rabbit feces with a higher identification rate for *Enterococcus* spp. strains (210/247, 85%) compared to *E. coli* strains (75/247, 30.4%), indicating the dominant presence of *Enterococcus* spp. in the fecal flora under local farming conditions. However, this lower recovery rate, possibly attributed to the reduced bioactivity of *E. coli* during feces processing and cell culture, should also be considered when compared with high isolation rates (57.1~100%) observed in other rabbit populations (19–21). Despite this discrepancy, an adequate number of *E. coli* and *Enterococcus* spp. strains were obtained for subsequent analysis.

We conducted an investigation on antibiotic resistance and relevant ARG distribution among these bacteria, and evaluated their susceptibility to seven available antimicrobials in two rabbit farms. A significant proportion of *E. coli* strains exhibited resistance to most antimicrobials (6/7) with diverse AMP spectrums, in contrast to *Enterococcus* spp. strains (3/7). This observation supports the previous finding that *Enterobacter* spp. harbor more mobile genetic elements (MGEs), facilitating robustly horizontal transfer of ARGs and acquisition of antibiotic resistance against specific antimicrobials (33). Notably, most strains of *E. coli* demonstrated reduced susceptibility to these antimicrobials, suggesting the need to consider alternative antibiotic prescription in light of the prevalence of DOX and AMP-resistant phenotypes. In addition, it is worth mentioning that no *E. coli* strain demonstrated resistance to PMB, which may be associated with less frequent medication. Although oral administration of ampicillin and amoxicillin/clavulanate may pose a risk of antibiotic-associated dysbiosis in certain rabbit populations (5), parenteral administration is still recommended for susceptible pathogens (18, 20). Moreover, antibiotics from the penicillin family appear to be more readily available for veterinary use in local rabbit farming practices, which may also influence the choice of antibiotic prescription and the prevalence of relevant antimicrobial resistance. Similarly, the frequent use of DOX and the sporadical use of PMB as informed by farm owners may influence the antibiotic susceptibility of *E. coli* strains.

Interestingly, most *Enterococcus* spp. strains only exhibited resistance to DOX and GEN, while no resistant strains against other antimicrobials were found. This disparity between *Enterobacter* spp. and *Enterococcus* spp. may be associated with their distinctive evolutionary pathways in antibiotic resistance against specific antimicrobials (34, 35). In particular, the difference in AMR spectrums between two farms was evident, possibly reflecting the preference of antibiotic prescriptions for veterinary technicians in farming practices, although the detailed medical history of antibiotic use was not comprehensively evaluated. Furthermore, bacteria of young rabbits origin exhibited a higher prevalence of antibiotic resistance compared to their adult counterparts, indicating that early-life exposure to antibiotics may robustly contribute to the emergence of antibiotic resistance among commensal bacteria (36). Therefore, reducing the inappropriate use of antimicrobials and medication overload will be a sustainable meat-rabbit farming practice.

According to the MIC values tested for resistant bacteria, increased levels of antibiotic resistance was prevalent, which may be inferred to the substantial exposure to antibiotics for rabbits in farming environments (30, 37). Furthermore, all GEN-resistant *Enterococcus* spp. strains showed high-level gentamicin resistance, whereas this characteristic was absent in GEN-resistant *E. coli* strains. This implies that *Enterococcus* spp. may serve as an active reservoir for acquired GEN resistance, which has also been detected in European wild rabbits (19). Previous research studies have indicated that patients with HLGR *Enterococcus* spp. strains of hospital origin could increase the risk of nosocomial infections, particularly the *E. faecalis* strains that typically harbor the aac[6']-Ie-aph[2”]-Ia gene (38, 39), and even produce cross-resistance with other critical important antibiotics (CIAs), such as vancomycin (40). It is important to note that the vancomycin-resistant *Enterococcus* spp. have been confirmed in food-producing animals (14, 15). Thus, increasing public attention should be paid toward these opportunistic pathogens of animal origin.

Several surveillance studies have reported a high prevalence of multi-resistant *E. coli* strains in farmed rabbits (10, 19–21). However, a similar occurrence (35/66, 53.0%) was still observed in our healthy rabbits. Additionally, we also identified a proportion of double-resistant *Enterococcus* spp. strains (40/164, 24.4%). As expected, most of multi-resistant *E. coli* strains carried ARGs associated with β-lactams (*blaTEM*, 34/35, 97.1%), tetracyclines (*tetA*, 33/35, 94.3%), fluroquinoloncs (*qnrS*, 25/35, 71.4%), and chloramphenicols (*floR*, 20/35, 57.4%) resistance. On the other hand, double-resistant *Enterococcus* spp. strains predominantly harbored the *aac[6']-Ie-aph[2”]-Ia* gene (31/40, 77.5%). Similar ARG distribution among resistant *E. coli* or *Enterococcus* spp. has been also found in farmed or wild rabbits, which may be attributed to environmental antibiotics co-selection (9, 15, 19, 41). Particularly, some resistant bacteria only carried a small number of ARGs (for example, *fexA, qnrD, tetB, tetM*, and *aac[6*′*]-lb*) or none at all, suggesting that certain ARG-independent mechanisms for antimicrobial resistance may be implicated for these bacteria (34, 35). It is well known that co-harboring various ARGs is one of the most important mechanisms to acquire antibiotic resistance (1, 12, 13); thus, the wide spread of antibiotic-specific ARGs among commensal *E. coli* and *Enterococcus* spp. strains may confer their resistance against commonly used antimicrobials, although other strain-level mobile genetic elements, including transposons, insertion sequences, and integrons (42), have not been evaluated in current study. In general, the commensal bacteria bearing abundant ARGs may serve as a high-risk reservoir of transferable antibiotic resistance, which pose unintended threats to rabbit welfare, farming biosecurity, and public health in a “One Health” perspective.

One limitation of the current study is the unexpanded AMR spectrum analysis for other groups of antibiotics, such as cephalosporins (e.g., ceftiofur), macrolides (e.g., azithromycin), sulfonamides (e.g., sulfadiazine/trimethoprim), and tricyclic glycopeptides (e.g., vancomycin), although they are rarely or never used in local rabbit farming practices. Second, the influence determinants involving antibiotic usage and consumption, bacteria genetic lineages, and biosecurity measures (15, 30, 31, 37) should be correlated with the emergence and enhancement of antibiotic resistance in further study. Even so, our findings provided a novel perspective on the impact of commonly used antimicrobials in shaping distinctive AMR spectrums and the increasing level of antibiotic resistance among indicator bacteria in meat-rabbit farming practices.

## 5 Conclusion

Overall, the distribution of AMR and ARGs is prevalent in healthy meat-rabbits, and the use of therapeutic antimicrobials in farming practices may promote the antibiotic resistance transmission among indicator bacteria. Therefore, periodic surveillance of antibiotic resistance in different geographic locations and supervisory measures for rational antibiotic use are considered to be imperative strategies for combating the rising threats posed by antibiotic resistance and maintaining rabbit welfare and public health.

## Data availability statement

The raw data supporting the conclusions of this article will be made available by the authors, without undue reservation.

## Author contributions

CS: Writing – original draft, Writing – review & editing, Formal analysis. ZW: Investigation, Writing – original draft, Writing – review & editing. YL: Formal analysis, Methodology, Writing – review & editing. JH: Conceptualization, Funding acquisition, Writing – review & editing.
