# Supplementary material for: Antibiotic resistance spectrums of Escherichia coli and Enterococcus spp. strains against commonly used antimicrobials from commercial meat-rabbit farms in Chengdu City, Southwest China
Source: Front Vet Sci. 2024 May 2;11:1369655. doi: 10.3389/fvets.2024.1369655 (PMC11096573; doi:10.3389/fvets.2024.1369655)
Supplement: Supplementary file 1 [file Data_Sheet_1.PDF]

Supplementary Material

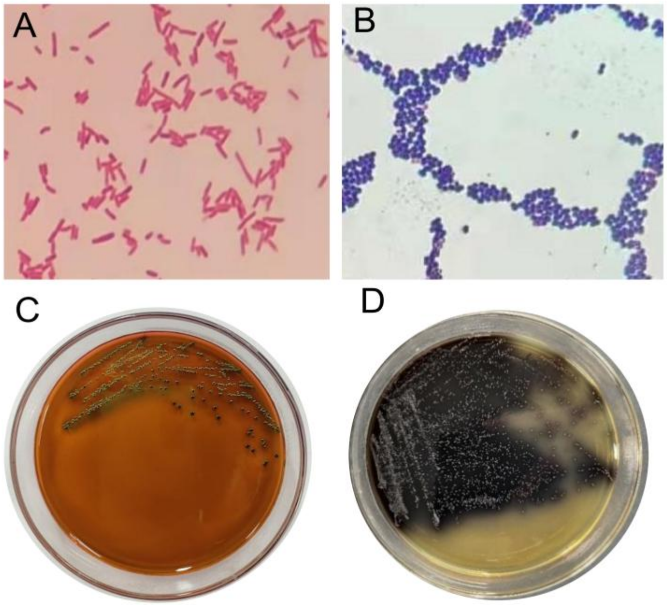

Supplementary Figure 1. Microscopic and gross morphology of suspected *E. coli* and *Enterococcus* spp. isolates. The Gram-negative *E. coli* appeared to be rod-like bacillus and formed deep purplish-black colonies with metallic sheen in EMB agar (A and C). The Gram-positive *Enterococcus* spp. appeared to be spherical and formed black colonies on *Enterococci* differential medium (B and D).

Supplementary Table 1. PCR primer sequences for ARGs detection

ARGs	Primer sequence (5’-3’)	Fragment size(bp）	Source
blaTEM	F: CAGCGGTAAGATCCTTGAGA<br>R: ACTCCCCGTCGTGTAGATAA	734	[9]
tetA	F：GGTTCACTCGAACGACGTCA<br>R：CTGTCCGACAAGTTGCATGA	577	[25]
tetB	F：CCTCAGCTTCTCAACGCGTG<br>R：GCACCTTGCTGATGACTCTT	634	[25]
tetM	F：ATAGAYACGCCAGGMCATA<br>R：GGAGCCCAGAAAGGATTYGG	1070	[25]
qnrS	F：TATTAACGGGCTGGCATTT<br>R：AGCTGTCAGGTGGGTCAAAC	417	[26]
qnrD	F：GTTTTCACGAGATCAATTTACGG<br>R：GTGAACAATAACACCTAAACTCTC	613	[26]
fexA	F：GCTCGGTGGTATCTCTGCTC<br>R：CAAAGCCCCTTGCTTGTTAC	672	[9]
floR	F：CACGTTGAGCCTCTATAT<br>R：ATGCAGAAGTAGAACGCG	868	[9]
aac[6′]-Ib	F：TTGCGATGCTCTATGAGTGGCTA<br>R：CTCGAATGCCTGGCGTGTTT	482	[27]
aac[6′]-Ie-aph[2″]-Ia	F：CAGGAATTTATCGAAAATGGTAGAAAAG<br>R：CACAATCGACTAAAGAGTACCAATC	369	[27]
